# Prevalence, clinical characteristics, and risk factors of intracerebral haemorrhage in CADASIL: a case series and systematic review

**DOI:** 10.1007/s00415-023-12177-0

**Published:** 2024-01-13

**Authors:** Nontapat Sukhonpanich, Hugh S. Markus

**Affiliations:** 1 Stroke Research Group, Department of Clinical Neurosciences, University of Cambridge, Cambridge Biomedical Campus, Cambridge, CB2 0QQ UK; 2 Department of Medicine, Faculty of Medicine Siriraj Hospital, Mahidol University, Bangkok, Thailand

**Keywords:** CADASIL, Intracerebral haemorrhage, ICH, Cerebral small vessel disease, Case series, Systematic review

## Abstract

**Background:**

Cerebral autosomal dominant arteriopathy with subcortical infarcts and leukoencephalopathy (CADASIL) is the most common monogenic form of stroke and is characterised by early onset stroke and dementia. Most strokes are lacunar ischaemic strokes, but intracerebral haemorrhage (ICH) has also been reported, although there are limited published data on its frequency and characteristics.

**Methods:**

A retrospective review of a prospectively recruited CADASIL register from the British National Referral clinic was performed to identify acute ICH cases and their characteristics. In addition, a systematic review of ICH in CADASIL was performed. MEDLINE (Pubmed), Embase, and Web of Science were searched for articles published from inception until 31/05/2023.

**Results:**

Ten cases of ICH were identified from the National clinic register of 516 symptomatic patients, giving an estimated point prevalence of 1.9%. An additional 119 cases were identified from the systematic review, comprising 129 cases and 142 ICH events in total. Including all identified cases, the mean age at onset of ICH was 56.6 ± 15.7 (SD) years, and 74 (57.4%) were male. ICH was the first manifestation of the disease in 32 patients (38.1%), and ICH recurrence occurred in 16 (12.4%). Most ICHs were subcortical, with the thalamus, 58 (40.8%), and basal ganglia, 34 (23.9%), being the commonest sites. Anticoagulation, but not antiplatelet agents, was associated with an increased risk of ICH (20.0% vs. 1.9%, *p* = 0.006).

**Conclusions:**

ICH is a relatively rare manifestation of CADASIL, occurring in about 2% of symptomatic cases. Most of the haemorrhages occurred in the subcortical regions.

**Supplementary Information:**

The online version contains supplementary material available at 10.1007/s00415-023-12177-0.

## Introduction

Cerebral autosomal dominant arteriopathy with subcortical infarcts and leukoencephalopathy (CADASIL) is the most common monogenic form of stroke, characterised by the early onset of ischaemic stroke and dementia. It is caused by a mutation in the *NOTCH3* gene, which usually changes the number of cysteine residues within the extracellular epidermal growth factor-like repeats (EGFr) in the NOTCH3 receptor [1]. Common manifestations of CADASIL include stroke, usually an ischaemic stroke of lacunar subtype, migraine with aura, psychiatric syndromes, vascular cognitive impairment, and dementia.

Until recently, intracerebral haemorrhage had been little described in CADASIL. Several ICH cases in CADASIL have been reported, especially in Asian populations [2]. However, the published data on the prevalence and characteristics of ICH in CADASIL are still limited. Therefore, to better understand ICH in CADASIL, we identified cases of ICH in a prospective CADASIL register to determine its prevalence and characteristics. We also performed a systematic review to identify previously published cases and analysed clinical features, risk factors, and outcomes in our case series combined with the previously published cases.

## Methods

### Case series

#### Data acquisition

We performed a retrospective review of a prospectively collected CADASIL register from the CADASIL National Referral clinic in Cambridge (from 2001 to 2023) and from the UK Familial Small Vessel Disease Study (from 2016 to 2023), which has been recruiting patients with monogenic small vessel disease (SVD) from 6 stroke centres in the UK. Patients’ data were collected at the first study encounter and follow-up visits using standardized forms. All CADASIL patients were diagnosed with a typical cysteine-changing *NOTCH3* mutation, and only symptomatic patients were included in the analysis. ICH was defined as an acute neurological syndrome with a confirmed presence of intraparenchymal blood by either computed tomography (CT) or magnetic resonance imaging (MRI).

All available imaging studies were reviewed to identify evidence of ICH lesions, complications, and radiological changes, including white matter lesions, lacunes, and cerebral microbleeds (CMBs). As per STRIVE criteria [3], lacune was defined as a subcortical infarct between 3 and 15 mm in diameter. CMB was defined as a small area, not larger than 10 mm in diameter, of signal void with associated blooming seen on T2*-weighted or susceptibility-weighted MRI sequences. Scans at the time of ICH were available in 7 patients and were graded individually. ICH details of the remaining 3 were extracted from the original radiology reports and, in addition, in a post-acute gradient echo sequence in one (A6).

### Statistical analysis

Categorical variables were presented in the number of cases and percentage (%), and continuous variables were displayed by mean and standard deviation (SD). Baseline characteristics between CADASIL patients with and without ICH were compared using Student’s *t* test and Pearson’s chi-squared test as appropriate. A *p* value of less than 0.05 was considered significant. An estimated point prevalence of ICH in CADASIL patients was calculated in percentage (%) within the prospective cohort. All analyses were carried out using the R software version 4.3.1.

## Systematic review

### Search strategy

The study protocol was pre-registered on PROSPERO (CRD42023425877), provided by the University of York, United Kingdom. We followed the Preferred Reporting Items for Systematic Reviews and Meta-Analyses (PRISMA) Statement guidelines, 2020 [4]. MEDLINE (Pubmed), Embase, and Web of Science were searched to identify studies published from inception until 31/05/2023. Search terms included "cerebral autosomal dominant arteriopathy with subcortical infarcts and leukoencephalopathy" OR "CADASIL", AND, "hemorrhage" OR "hemorrhages" OR "haemorrhage" OR "haemorrhages" OR "bleed", and were refined according to each database. In addition, reference lists of the relevant articles were also searched.

### Study selection and eligibility criteria

Studies were deemed eligible if they met the following criteria: (1) CADASIL diagnosed by one of the following criteria: confirmed *NOTCH3* mutation, the presence of granular osmiophilic material (GOM) from skin biopsy, or known family mutation with clinical features; (2) original human data; and (3) availability of acute ICH details from patients’ history or scans. Asymptomatic haemorrhagic lesions were not included. Quality was assessed using the Joanna Briggs Institute (JBI) critical appraisal checklists [5, 6]. Regardless of language, all studies that fulfilled the criteria were included. In case there was a potential of data overlapping between studies, we contacted the corresponding authors to get the original patient data.

### Data extraction and analysis

Demographic data extracted included age, sex, mutation detail, history of any CADASIL features, radiographic changes due to CADASIL, vascular risk factors, and antithrombotic medication used. Regarding the ICH detail, clinical features, radiological characteristics, and location of ICH were extracted. The cases, including demographics, risk factor details, and clinical and radiographic features identified from the systemic review, were pooled with the UK case series for the combined analysis.

## Results

### Case series

A total of 544 CADASIL patients were identified from the register. Of these, 516 were symptomatic and had typical heterozygous cysteine-changing mutations and were included in the analysis. We excluded 26 who were asymptomatic and diagnosed on predictive testing, one with a compound heterozygous mutation, and one with a homozygous mutation. The data from 516 symptomatic CADASIL patients were available, of whom ten patients had suffered from ICH, giving an ICH point prevalence of 1.9%. One patient also suffered one ICH recurrence. The mean age at ICH was 53.3 ± 7.6 (SD) years. In two cases, ICH was the presenting feature of CADASIL. There was no difference in the proportion of those with a mutation in EGFr 1–6, which was previously associated with increased ischaemic stroke risk [7], between those with and without ICH (50.0% vs. 79.4%, *p* value = 0.112). In those cases with ICH, there was a nonsignificant trend to a higher history of hypertension (50.0% vs. 23.9%, *p* value = 0.126), and a significant increase of anticoagulant use (20.0% vs. 1.9%, *p* value = 0.006), but not of antiplatelet agents. Details of individual patients with ICH are shown in Table 1. Patients’ mutation details and differences in demographic and clinical features of those with and without ICH are available in the online resources (Table S1, S2).

**Table 1:** Summary of clinical and radiological characteristics of individual CADASIL patients with ICH in the UK cohort

Case	Age^a^	Sex	Previous CADASIL feature	ICH symptom	Recovery^b^	ICH Location	Size (cm)	IVH	Imaging marker		Vascular risk	Antithrombotic
									CMB	Lacune		
A1	60	F	Migraine, encephalopathy, IS	Headache, dysphasia, decreased level of consciousness	Good	Lt. caudate	3.0 × 2.5	+	0	3	Ex-smoking	Aspirin
A2	60	M	Migraine	Headache, vomiting, left-sided weakness	Good	Rt. basal ganglia	1.9 × 2.1	+	3	7	HLP, ex-smoking	Clopidogrel
A3	58	F	Migraine, cognitive impairment	Confusion	Poor	Rt. thalamus	1.3 × 1.1	−	> 80	12	HT	Aspirin
A4	47	M	IS	Headache, left leg weakness	Good	Rt. superior parietal WM	1.4 × 1.3	−	0	5	HT, DM type 1, ex-smoking	Clopidogrel
A5	57	F	None	Collapse, slurred speech, left-sided weakness	Good	Rt. thalamus	2.3 × 1.7	+	0	1	None	None
A6	42	F	Depression, cognitive impairment, migraine	Left-sided weakness	Good	Rt. thalamus	N/A	+	6	3	HT	Clopidogrel
A7	51	M	Depression, TIA	Confusion, dysphasia, seizure	Poor	Lt. medial temporal cortex	1.5 × 2.1	−	2	0	HT, HLP, ex-smoking	Aspirin
				Decreased level of consciousness, right facial droop, vomiting	Deceased	Lt. medial temporal cortex	2.9 × 6.7	+				None^c^
A8	61	M	None	Headache, vomiting, slurred speech, ataxia	Good	Cerebellar vermis	3.9 × 1.2	−	0	1	None	None
A9	41	F	Migraine, IS, cognitive impairment	Right-sided weakness, dysphasia	Deceased	Brainstem^d^	N/A	-	N/A	N/A	None	Anticoagulant
A10	56	M	IS	Visual disturbance	NA	Rt. medial temporal cortex^d^	N/A	−	N/A	N/A	HLP	Aspirin, warfarin

*CADASIL* cerebral autosomal dominant arteriopathy with subcortical infarcts and leukoencephalopathy, *CMB* cerebral microbleed, *DM* diabetes mellitus, *HLP* hyperlipidaemia, *HT* hypertension, *ICH* intracerebral haemorrhage, *IS* ischaemic stroke, *IVH* intraventricular haemorrhage, *N/A* not applicable, *TIA* transient ischaemic attack

^a^Age when the patient had ICH was used

^b^A patient with good or poor recovery was defined as having a mRS of 0–1 or mRS ≥ 2, respectively

^c^Aspirin was already stopped after his first haemorrhage

^d^ICH details of these patients were extracted from the original reports

Six patients recovered to a modified Rankin score (mRS) of less than 2, one had a mRS of 4, while two died from the ICH. Outcome details were not available in one patient (A10). One patient (A7) had a recurrent ICH at the left medial temporal cortex, occurred 14 days after he had a brain biopsy at that region due to the suspicion of a primary brain tumour, which showed gliotic changes.

**Fig. 1:**
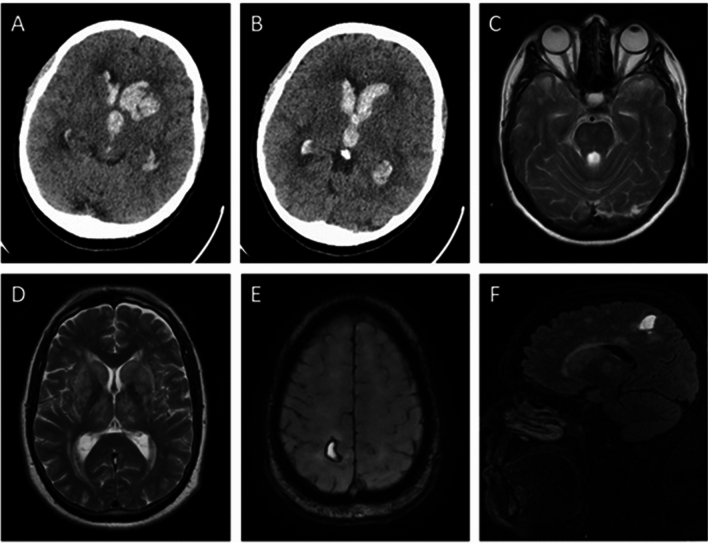
**A**–**C** Brain imaging findings of patient A1. A non-contrast CT scan **A**, **B** revealed a left caudate haemorrhage with intraventricular extension. T2-weighted MRI sequences **C** demonstrated diffuse WMH with bilateral temporal pole involvement. **D**–**F** Brain imaging findings of patient A4. T2-weighted MRI **D** demonstrated diffuse WMH involving bilateral external capsules. Susceptibility-weighted **E** and T1-weighted **F** MRI sequences revealed ICH at the subcortical white matter of right superior parietal lobule

On brain imaging, the location of the ICH was thalamic in 3 patients, caudate in 1, basal ganglia in 1, medial temporal cortex in 3, subcortical white matter in 1, cerebellum in 1, and brainstem in 1. Intraventricular extension was observed in 5 (45.5%), most of which were deep ICHs. Figure 1 shows examples of imaging characteristics of ICH in two patients. Out of the 8 patients with original gradient echo or susceptibility scans available for review, subcortical and/or infratentorial CMBs were present in 4 patients.

### Systematic review

**Fig. 2:**
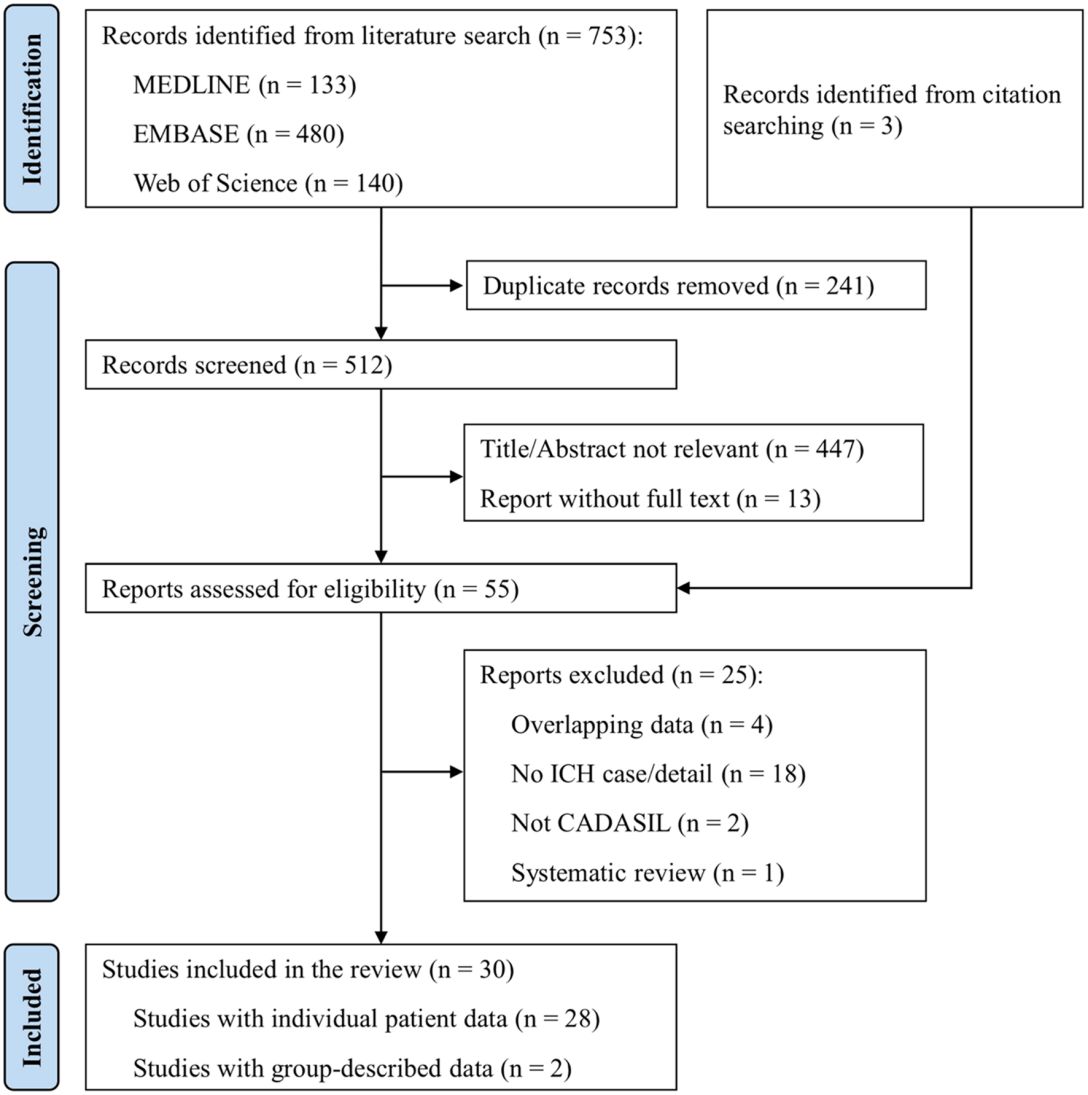
PRISMA flow diagram of the searching strategy and study selection

Through the search, a total of 756 articles were identified. Fifty-five articles were eligible for full-text assessment and 25 were excluded for four reasons, summarised in Fig. 2. We contacted the corresponding authors regarding possible data overlapping issues in 8 articles [8–15]. Following this, we included 4 of them, two which had been confirmed to have no overlapping data [11, 15] and for 2 where there was overlap but we were provided with the individual patient data [13, 14]. We received no reply for one potential overlap [8–11] so we omitted smaller studies from this group [8–10] and only included the larger study [11].

Thirty studies were eligible, including 21 case reports [16–36], 7 case series [13, 14, 37–41], and 2 observational studies [11, 15], comprising 119 CADASIL patients with history of ICH. Half of the studies were published in Asia, which consisted of 98 cases (76.0%). The details of each study are summarised in Table 2. The details of the quality assessment and mutation and diagnostic means in each study are available in the online resources (Table S3–S6).

**Table 2:** Summary of 30 studies included in the systematic review (119 cases)

Study	No. of cases	Age^a^	Sex^b^	ICH location	Died from ICH	Imaging marker		Vascular risk	Antithrombotic
						Lacune	CMB		
Sourander et al. [16]	1	29	M	Thalamus	Yes	−	N/A	–	Anticoagulant
Baudrimont et al. [17]	1	59	F	Basal ganglia and thalamus	Yes	+	N/A	HLP	N/A
MacLean et al. [18]	1	56	M	Subcortical WM	Yes	N/A	+	Smoking	–
Ragoschke-Schumm et al. [19]	1	47	F	Cerebellum	No	+	+	HT	–
Werbrouck and De Bleecker [20]	1	45	M	Basal ganglia	No	+	+	HT, HLP	Warfarin
Kotorii et al. [21]	1	72	F	–	No	+	+	–	–
Choi et al. [13]	5	64.4	M (3) F (2)	Basal ganglia (3), Thalamus (2), Cerebellum (1)	No	+ (5)	+ (5)	HT (5), DM (1), HLP (1)	Antiplatelet (3)
Oh et al. [22]	1	39	M	Temporal cortex	No	+	+	HT, smoking	Aspirin
Mizuno et al. [23]	1	64	F	Thalamus	No	+	N/A	HT, DM	N/A
Lee et al. [37]	4^c^	53.5	M (3) F (1)	Thalamus (2), Basal ganglia (1), Subcortical WM (1), Parietal lobe^d^ (1)	No	+ (3)	N/A	HT (4)	Aspirin (3)
Delgado et al. [24]	1	55	M	Subcortical WM	No	N/A	N/A	–	–
Sano et al*.* [25]	1	46	M	Basal ganglia	No	N/A	+	smoking	Ticlopidine
Pradotto et al. [26]	1	65	M	Subcortical WM	No	N/A	+	–	LMWH
Lian et al. [27]	1	46	M	Basal ganglia	No	+	+	HLP, smoking	–
Mehta et al. [28]	1	55	F	Cerebellum	No	+	+	–	–
Rinnoci et al. [29]	3	67.3	M (2) F (1)	Thalamus (3), Cerebellum (1)	No	+ (3)	+ (3)	HT (3), smoking (2)	–
Choi et al. [14]	4^c^	51.8	M (2) F (2)	Basal ganglia (4)	No	+ (3)	+ (4)	HT (3), DM (1), smoking (1)	Antiplatelet (2)
Marlen et al. [30]	1	47	M	Occipital cortex	No	+	+	HT	Aspirin
Koutroulou et al. [31]	1	30	M	Thalamus	No	−	+	–	–
Zhang et al. [32]	2	60.5	M (1) F (1)	Thalamus (1), Parietal cortex (1), Subcortical WM (1), Temporal lobe (1)	No	+ (2)	+ (2)	HT (1), smoking (1)	–
Bersano et al. [38]	1	61	M	Basal ganglia	No	N/A	N/A	N/A	N/A
Chiang et al. [33]	1	57	M	Pons	Yes	+	+	HT	Aspirin
Kim et al. [39]	3	54	M (3)	Thalamus (2), Basal ganglia (1)	No	N/A	+ (2)	HT (1)	N/A
Wang et al. [34]	1	38	M	Subcortical WM	No	+	+	HT	N/A
Palazzo et al. [40]	5	45.6	M (4) F (1)	Parietal cortex (1), Thalamus (2), Basal ganglia (1), Subcortical WM (1)	Yes (1)	+ (5)	+ (4)	HT (3), HLP (2), smoking (1)	Aspirin (2)
Liao et al. [15]	27^c^	58.9	M (12) F (15)	Thalamus (12), Basal ganglia (3), Corona radiata (2), Subcortical WM (2), Occipital cortex (1), Medial temporal cortex (1) Cerebellum (4), Pons (4)	No	N/A	+ (15)	HT (20)	Aspirin (9), Clopidogrel (3), Cilostazol (1), Aspirin + dipyridamole (1) Warfarin (1)
Hu et al. [35]	1	60	M	Cerebellum (2), Basal ganglia (1)	No	+	+	HT	Clopidogrel
Chen et al. [11]	45	59.1	M (23) F (22)	Thalamus (25), Basal ganglia (11), Cortico-subcortical (5), Brainstem (2), Cerebellum (1), Multiple (1)	Yes (4)	+ (43)	+ (45)	HT (39), DM (12), HLP (17), smoking (11)	N/A
Chu et al. [36]	1	59	M	Thalamus (3), Basal ganglia (1)	No	+	+	−	–
Nogueira et al. [41]	1	40	M	Basal ganglia	No	+	+	−	Aspirin

*CMB* cerebral microbleed, *DM* diabetes mellitus, *F* female, *HLP*, hyperlipidaemia, *HT* hypertension, *ICH* intracerebral haemorrhage, *LMWH* low molecular weight heparin, *M* male, *N/A* not applicable, *No* number, *WM* white matter

^a^Age when the patient had ICH was used. Studies with more than one value were shown in mean

^b^The number in the parenthesis indicates the number of cases

^c^Some patients from these studies were excluded due to having only asymptomatic haemorrhage

^d^The exact location of ICH wasn’t mentioned

### Combined analysis of case series and systematic review of cases

Combining the result with our case series, 129 CADASIL patients with 142 ICH events were identified. Data on genetic testing were available in 124 (96.1%). CADASIL was diagnosed on the basis of a *NOTCH3* mutation in 123 (95.3%), a known family mutation with clinical features in the absence of genetic confirmation in 2 (1.6%) [13, 40], GOM in the absence of genetic testing in 3 (2.3%) [16, 17, 30], and GOM in 1 patient with negative testing in exon 2–24 [19], Of those with a diagnosed *NOTCH3* mutation, 119 (96.7%) were typical cysteine altering, 3 (2.4%) were cysteine sparing, and 1 with genetically confirmed CADASIL but *NOTCH3* mutation detail wasn’t mentioned [33].

A summary of the characteristics of all patients is shown in Table 3. The mean age at the time of ICH was 56.6 ± 15.7 (SD), and 74 patients (57.4%) were male. Asian CADASIL patients had ICH at an older mean age compared to other regions (58.1 ± 15.4 and 51.9 ± 10.6 years, respectively) (online resources, Table S7). Of 84 patients with available information, 32 (38.1%) had ICH as the first manifestation of CADASIL.

**Table 3:** Summary of characteristics of all CADASIL patients with ICH, including cases from our cohort (129 patients)

Characteristics^a^	All cases (*n* = 129)
Age at ICH; y, mean ± SD	56.6 ± 15.7
Male	74 (57.4%)
Study site of reported cases	
Asia	98 (76.0%)
Europe	26 (20.2%)
North America	3 (2.2%)
South America	2 (1.6%)
Diagnosis of CADASIL^b^	
Cysteine-changing *NOTCH3* mutation	119 (92.2%)
Cysteine-sparing *NOTCH3* mutation	3 (2.3%)
GOM	4 (4.6%)
Known family mutation with clinical features	2 (1.6%)
CADASIL feature	
ICH as the first manifestation	32/84 (38.1%)
Previous ischaemic stroke	42/129 (32.6%)
Previous migraine	10/71 (14.1%)
Previous cognitive impairment	15/71 (21.1%)
Vascular risk factors	
Hypertension	91/128 (71.1%)
Diabetes mellitus	16/101 (15.8%)
Hyperlipidaemia	26/101 (25.7%)
History of smoking	24/94 (25.5%)
Antithrombotic medication	
Antiplatelet^c^	37/77 (48.1%)
Aspirin	22/32 (68.8%)
Clopidogrel	7/32 (21.9%)
Anticoagulant	6/77 (7.8%)
Warfarin	3/4 (75.0%)
LMWH	1/4 (25.0%)
Symptom	
Headache	12/37 (32.4%)
Altered mental status	13/37 (35.1%)
Weakness	22/37 (59.5%)
Dysphasia	10/37 (27.0%)
Seizure	4/37 (10.8%)
Recurrent ICH	16/129 (12.4%)
Intraventricular extension	25/116 (21.6%)
Died from ICH	11/129 (8.5%)
ICH location (142 lesions)^d^	
Thalamus	58/142 (40.8%)
Basal ganglia	34/142 (23.9%)
Subcortical WM	18/142 (12.7%)
Cortical	10/142 (7.0%)
Cerebellum	12/142 (8.5%)
Brainstem	8/142 (5.6%)
Imaging characteristics	
Have CMB	99/105 (94.3%)
CMBs ≥ 10	66/84 (78.6%)
Have Lacune	86/93 (92.5%)

*CADASIL* cerebral autosomal dominant arteriopathy with subcortical infarcts and leukoencephalopathy, *CMB* cerebral microbleed, *GOM* granular osmiophilic material, *ICH* intracerebral haemorrhage, *WM* white matter

^a^Due to a variety of reported individual-level patient data in different studies, the denominators differed in each characteristic

^b^One patient was reported genetically confirmed CADASIL, but the mutation was not mentioned

^c^Other antiplatelets included cilostazol in 1 patient (3.1%), aspirin and dipyridamole in 1 patient (3.1%), and ticlopidine in 1 patient (3.1%)

^d^The exact location was not known in 2 cases. One case reported a parietal lobe ICH, but the exact region wasn’t mentioned. Another case reported multiple ICH lesions

Hypertension was the most common vascular risk factor present in 91 patients (71.1%) and was more commonly seen in Asian patients (78.6% vs. 46.7%). Six patients (7.8%) were on anticoagulants (warfarin in 3 patients, low molecular weight heparin (LMWH) in 1, and two, the unspecified type).

A total of 142 ICH lesions were identified from brain imaging or autopsy studies. Most lesions were subcortical haemorrhages (130, 91.5%). The sites of haemorrhages were thalamus (58, 40.8%), basal ganglia (34, 23.9%), subcortical white matter (18, 12.7%), cerebellum (12, 8.5%), brainstem (8, 5.6%), and cortical (10, 7.0%). One ICH was in the parietal lobe but was not specified if it was cortical or subcortical (1, 0.7%), and the remaining case was reported as having multiple lesions (1, 0.7%). This trend was similarly seen across different ethnicities. All patients with brain MRI (128, 99.2%) had widespread white matter hyperintensities. CMBs were present in 99 of 105 patients (89.3%) of whom information was reported, and lacunes 86 of 93 patients (92.5%).

Intraventricular extension was observed in 25 patients (21.6%), with most being basal ganglia or thalamic haemorrhages. Out of 129 cases, 11 patients (8.5%) died acutely from the ICH; of these, three patients had large basal ganglia or thalamic haemorrhages with intraventricular extension, two had brainstem haemorrhages, one had an extensive left temporal lobe haemorrhage with severe mass effect, one with right frontal subcortical haemorrhage developed aspiration pneumonia, and in the remaining four details were not specified.

## Discussion

In this study, we describe the point prevalence of ICH in a large prospectively recruited CADASIL cohort at about 2%. In a systematic review of published literature, we further identified 119 cases, giving a total of 129 patients with 142 ICHs when combined with our case series. This allowed us to characterise the characteristics of ICH in CADASIL. About 92% were subcortical, with the commonest locations being the thalamus (40.8%) and basal ganglia (23.9%), while 14.1% were infratentorial. Intraventricular extension occurred in 21.6%, and the ICH was fatal in 8.5%.

We observed a similar point prevalence of ICH to that previously reported from European CADASIL cohorts, which ranged from 0.5% to 2.7% [42–44]; however, ICH was more commonly observed in the Asian cohorts. A recent meta-analysis reported the pooled prevalence of ICH in CADASIL of 10.1%, with 17.7% and 2.0% within the Asian and European subgroups, respectively [45–47]. The mechanism leading to this difference is unknown, although the Asian population has been reported to have a higher prevalence of hypertension in CADASIL patients [48].

In our cohort, there was no difference in the proportion of patients with a mutation in the EGFr 1–6, which has been associated with an increased risk of ischaemic stroke [7], between those with and without ICH. Whether the mutation site is related to the ICH phenotype remains to be further explored. Interestingly, the mutation profiles in CADASIL patients are slightly different as most of the mutations in Far East Asia occur in exon 11 [45, 47], while in our cohort, mutations mostly occur in exon 4 (63.6% in the whole cohort and 40.0% in ICH patients) (online resource Table S2).

We also describe the risk factor profile in patients with ICH, although a comparison group without ICH was only available in our case series. Hypertension was the most common vascular risk factor present in 71.1%. The prevalence was higher than those observed in large CADASIL cohorts (23.0%–39.4%) [44, 45, 49, 50]. Hypertension has been associated with an increased risk of ischaemic stroke in CADASIL [12] and has been suggested as a risk factor for ICH in CADASIL in previously published studies [15, 51]. Our results suggest that CADASIL is associated with ICH rather than occurring independently in patients with risk factors such as hypertension. In support of this, our observed ICH point prevalence at 2% is considerably higher than the ICH prevalence rate one would expect in the UK population (about 185.1 cases per 100,000 or 0.2%) [52]. Furthermore, about half of the ICH patients in our cohort did not have hypertension at the time of ICH. About half (48.1%) were prescribed antiplatelet during the time of ICH, and 7.8% were prescribed anticoagulants. In our case series, there was a significant increase in ICH in patients taking anticoagulants but not antiplatelets. Concern over this risk of ICH has led European Guidelines to suggest anticoagulants should be avoided in CADASIL, unless there is an alternative reason why they must be given [53].

CMBs were also common in CADASIL cases with ICH, occurring in more than 90% of those with appropriate MRI sequences performed. CMBs are frequently present in CADASIL, being reported in 31–66% [51, 54], but the figure we found seems higher.

Our study has a number of strengths. The case series was identified from large, prospectively recruited CADASIL patients, making it possible to estimate point prevalence. Furthermore, we identified all other published cases from the literature. However, it also has limitations. The CADASIL cohort only included patients presenting to the UK National CADASIL clinic and other clinical stroke services, so there may be a referral bias. Owing to the rarity of ICH in CADASIL, most included studies were case reports, and there was no control group for the systematic review cases. Therefore, it wasn’t possible to reliably determine the association between the potential risk factors and ICH. In this review, three patients from Asian studies had a cysteine-sparing *NOTCH3* mutation. It is still controversial about the pathogenicity of these mutations in CADASIL, which might be different from typical CADASIL.

In conclusion, ICH is rare in CADASIL but does occur in about 2% of individuals. It is usually subcortical, appears more common in Far East Asia, and may be associated with hypertension and anticoagulants.

### Supplementary Information

Below is the link to the electronic supplementary material.

Supplementary file1 (DOCX 51 KB)

## Data Availability

The anonymised data are available from the corresponding author upon reasonable request.
