# Supplementary material for: Prevalence, clinical characteristics, and risk factors of intracerebral haemorrhage in CADASIL: a case series and systematic review
Source: J Neurol. 2024 Jan 13;271(5):2423–33. doi: 10.1007/s00415-023-12177-0 (PMC11055697; doi:10.1007/s00415-023-12177-0)
Supplement: Supplementary file 1 — Supplementary file1 (DOCX 51 KB) [file 415_2023_12177_MOESM1_ESM.docx]

**Supplementary Materials**

**Prevalence, Clinical Characteristics, and Risk Factors of Intracerebral Haemorrhage in CADASIL – Case Series and Systematic Review**

Nontapat Sukhonpanich, MD^1,2^, Hugh S. Markus, DM, FMed Sci^1^

^1^Stroke Research Group, Department of Clinical Neurosciences, University of Cambridge, Cambridge, United Kingdom

^2^Department of Medicine, Faculty of Medicine Siriraj Hospital, Mahidol University, Bangkok, Thailand

**Corresponding author:** Hugh S. Markus, DM, FMed Sci

Email: [hsm32@medschl.cam.ac.uk](mailto:hsm32@medschl.cam.ac.uk)

**Table S1** Summary of mutation details of CADASIL patients with ICH in our cohort

| Case | Ethnicity | Nucleotide | Codon | Exon | EGFr domain | Protein | Cysteine-changing |
| --- | --- | --- | --- | --- | --- | --- | --- |
| A1 | White-British | c.544C>T | 182 | 4 | 4 | p.Arg182Cys | gain |
| A2 | White-British | c.733T>A | 245 | 5 | 6 | p.Cys245Ser | loss |
| A3 | White-British | c.1672C>T | 558 | 11 | 14 | p.Arg558Cys | gain |
| A4 | White-British | c.1345C>T | 449 | 8 | 11 | p.Arg449Cys | gain |
| A5 | White-British | c.1672C>T | 558 | 11 | 14 | p.Arg558Cys | gain |
| A6 | White-British | c.1547G>T | 516 | 11 | 13 | p.Cys516Phe | loss |
| A7 | White-British | c.1279C>T | 427 | 8 | 10 | p.Arg427Cys | gain |
| A8 | White-British | c.544C>T | 182 | 4 | 4 | p.Arg182Cys | gain |
| A9 | White-British | c.421C>T | 141 | 4 | 3 | p.Arg141Cys | gain |
| A10 | White-British | c.421C>T | 141 | 4 | 3 | p.Arg141Cys | gain |

Abbreviation: EGFr, epidermal growth factor-like repeat

**Table S2** Comparison of demographics, clinical features, and risk factors of CADASIL patients with ICH and without ICH in the UK Cohort

| **Characteristics^a^** | **ICH (n = 10)** | **No ICH (n = 506)** | **p-value** |
| --- | --- | --- | --- |
| Age at assessment; mean (SD) | 58.7 (10.2)^b^ | 60.9 (13.6) | 0.506 |
| Age at onset; mean (SD) | 28.1 (14.6) | 31.9 (14.7) | 0.429 |
| Female sex | 5 (50.0%) | 290 (57.3%) | 0.889 |
| Mutation in EGFr 1-6 | 5 (50.0%) | 402 (79.4%) | 0.112 |
| CADASIL feature | | | |
| Ischemic stroke | 4 (40.0%) | 237 (46.8%) | 0.894 |
| Migraine | 5 (50.0%) | 377 (74.5%) | 0.166 |
| Cognitive impairment | 3 (30.0%) | 201 (39.7%) | 0.760 |
| Psychiatric symptoms | 2 (20.0%) | 202 (39.9%) | 0.222 |
| Encephalopathy | 1 (10.0%) | 51 (10.1%) | 1.000 |
| Seizure | 0 (0.0%) | 50 (9.9%) | 0.590 |
| Vascular risk factor | | | |
| Hypertension | 5 (50.0%) | 121 (23.9%) | 0.126 |
| Diabetes mellitus | 1 (10.0%) | 31 (6.1%) | 1.000 |
| Hyperlipidaemia | 3 (30.0%) | 250 (49.4%) | 0.370 |
| History of smoking | 4 (40.0%) | 240 (47.4%) | 0.884 |
| Antithrombotic medication | | | |
| Antiplatelet | 7 (70.0%) | 338/475 (71.2%) | 0.804 |
| Aspirin | 5/7 (71.4%) | 185/338 (54.7%) | 0.310 |
| Clopidogrel | 2/7 (28.6%) | 143/338 (42.3%) | 0.729 |
| Anticoagulant | 2 (20.0%) | 9/475 (1.9%) | **0.006** |
| VKA | 1/2 (50.0%) | 5/9 (55.6%) | 0.238 |
| DOACs | 0/2 (0.0%) | 4/9 (44.4%) | 1.000 |
| Imaging markers^c^ | | | |
| Lacune | 7/8 (87.5%) | 179/267 (67.0%) | 0.404 |
| CMB | 4/8 (50.0%) | 81/180 (45.0%) | 1.000 |

Abbreviation: CADASIL, cerebral autosomal dominant arteriopathy with subcortical infarcts and leukoencephalopathy; CMB, cerebral microbleed; DOACs, direct oral anticoagulants; EGFr, epidermal growth factor-like repeat; ICH, intracerebral haemorrhage; VKA, vitamin K antagonist

^a^Missing data were present in some variables and were shown with distinct denominators.

^b^For patients with ICH, age at ICH was used.

^c^Due to the heterogeneity of different scanners from different centres, lacune and microbleed could not be graded in some patients.

**Table S3** Quality assessment of included case reports.

| Criteria  Case report | 1. Were patient’s demographic characteristics clearly described? | 2. Was the patient’s history clearly described and presented as a timeline? | 3. Was the current clinical condition of the patient on presentation clearly described? | 4. Were diagnostic tests or assessment methods and the results clearly described? | 5. Was the intervention(s) or treatment procedure(s) clearly described? | 6. Was the post-intervention clinical condition clearly described? | 7. Were adverse events (harms) or unanticipated events identified and described? | 8. Does the case report provide takeaway lessons? |
| --- | --- | --- | --- | --- | --- | --- | --- | --- |
| Sourander *et al*. (1997) | ✓ | ✓ | ✓ | ✓ | 🗶 | ✓ | N/A | ✓ |
| Baudrimont *et al*. (1993) | ✓ | ✓ | ✓ | ✓ | 🗶 | ✓ | N/A | ✓ |
| MacLean *et al*. (2005) | ✓ | 🗶 | ✓ | ✓ | 🗶 | ✓ | N/A | ✓ |
| Ragoschke-Schumm *et al*. (2005) | ✓ | ✓ | ✓ | ✓ | ✓ | ✓ | 🗶 | ✓ |
| Werbrouck and De Bleecker (2006) | ✓ | ✓ | ✓ | ✓ | ✓ | ✓ | ✓ | ✓ |
| Kotorii *et al*. (2006) | ✓ | 🗶 | ✓ | ✓ | 🗶 | ✓ | N/A | ✓ |
| Oh *et al*. (2008) | ✓ | ✓ | ✓ | ✓ | ✓ | ✓ | ✓ | ✓ |
| Mizuno *et al*. (2008) | ✓ | ✓ | 🗶 | ✓ | 🗶 | ✓ | N/A | ✓ |
| Delgado *et al*. (2011) | ✓ | ✓ | ✓ | ✓ | 🗶 | ✓ | N/A | ✓ |
| Sano *et al*. (2011) | ✓ | ✓ | ✓ | ✓ | ✓ | ✓ | ✓ | ✓ |
| Pradotto *et al*. (2012) | ✓ | ✓ | ✓ | ✓ | ✓ | ✓ | ✓ | ✓ |
| Lian *et al*. (2013) | ✓ | ✓ | ✓ | ✓ | ✓ | ✓ | ✓ | ✓ |
| Mehta *et al*. (2013) | ✓ | ✓ | ✓ | ✓ | 🗶 | 🗶 | N/A | ✓ |
| Rinnoci *et al*. (2013) | ✓ | ✓ | ✓ | ✓ | 🗶 | 🗶 | N/A | ✓ |
| Marlen *et al*. (2016) | ✓ | ✓ | ✓ | ✓ | ✓ | ✓ | 🗶 | ✓ |
| Koutroulou *et al*. (2016) | ✓ | ✓ | ✓ | ✓ | 🗶 | 🗶 | N/A | ✓ |
| Zhang *et al*. (2017) | ✓ | ✓ | ✓ | ✓ | ✓ | ✓ | ✓ | ✓ |
| Chiang *et al*. (2019) | 🗶 | ✓ | ✓ | 🗶 | ✓ | ✓ | ✓ | ✓ |
| Wang *et al*. (2020) | ✓ | ✓ | 🗶 | ✓ | 🗶 | ✓ | N/A | ✓ |
| Hu *et al*. (2022) | ✓ | ✓ | ✓ | ✓ | ✓ | ✓ | ✓ | ✓ |
| Chu *et al*. (2023) | ✓ | ✓ | ✓ | ✓ | 🗶 | 🗶 | N/A | ✓ |

Abbreviation: N/A, not applicable; ✓, yes; 🗶, no

**Table S4** Quality assessment of included case series

| Criteria  Case series | 1. Were there clear criteria for inclusion in the case series? | 2. Was the condition measured in a standard, reliable way for all participants included in the case series? | 3. Were valid methods used for identification of the condition for all participants included in the case series? | 4. Did the case series have consecutive inclusion of participants? | 5. Did the case series have complete inclusion of participants? | 6. Was there clear reporting of the demographics of the participants in the study? | 7. Was there clear reporting of clinical information of the participants? | 8. Were the outcomes or follow up results of cases clearly reported? | 9. Was there clear reporting of the presenting site(s)/clinic(s) demographic information? | 10. Was statistical analysis appropriate? |
| --- | --- | --- | --- | --- | --- | --- | --- | --- | --- | --- |
| Choi *et al*. (2006) | ✓ | ✓ | ✓ | ✓ | ✓ | ✓ | ✓ | 🗶 | ✓ | ✓ |
| Lee *et al*. (2009) | ✓ | ✓ | ✓ | N/A | N/A | ✓ | ✓ | 🗶 | ✓ | ✓ |
| Choi *et al*. (2013) | ✓ | ✓ | ✓ | ✓ | ✓ | ✓ | ✓ | ✓ | ✓ | ✓ |
| Bersano *et al*. (2018) | ✓ | ✓ | ✓ | ✓ | ✓ | ✓ | ✓ | 🗶 | ✓ | ✓ |
| Kim *et al*. (2019) | ✓ | ✓ | ✓ | ✓ | ✓ | 🗶 | ✓ | 🗶 | ✓ | ✓ |
| Palazzo *et al*. (2021) | ✓ | ✓ | ✓ | ✓ | ✓ | ✓ | ✓ | 🗶 | 🗶 | ✓ |
| Nogueira *et al*. (2023) | ✓ | ✓ | ✓ | ✓ | ✓ | ✓ | ✓ | 🗶 | ✓ | ✓ |

Abbreviation: N/A, not applicable; ✓, yes; 🗶, no

**Table S5** Quality assessment of included observational study

| Criteria  Observational  study | 1. Were the criteria for inclusion in the sample clearly defined?" | 2. Were the study subjects and the setting described in detail? | 3. Was the exposure measured in a valid and reliable way? | 4. Were objective, standard criteria used for measurement of the condition? | 5. Were confounding factors identified? | 6. Were strategies to deal with confounding factors stated? | 7. Were the outcomes measured in a valid and reliable way? | 8. Was appropriate statistical analysis used? |
| --- | --- | --- | --- | --- | --- | --- | --- | --- |
| Liao *et al*. (2021) | ✓ | ✓ | ✓ | ✓ | ✓ | ✓ | ✓ | ✓ |
| Chen *et* *al.* (2022) | ✓ | ✓ | ✓ | ✓ | ✓ | ✓ | ✓ | ✓ |

Abbreviation: N/A, not applicable; ✓, yes; 🗶, no

**Table S6** Summary of all mutation details and pathological results

| **Study** | **Study site** | **No. of cases** | **Age^a^** | **Sex** | **Mutation detail** | | | **Pathological study** |
| --- | --- | --- | --- | --- | --- | --- | --- | --- |
|  |  |  |  |  | **Exon** | **Protein** | **Cysteine change** |  |
| Sourander *et al*. (1997) | Sweden | 1 | 29 | M | N/A | N/A | N/A | Post-mortem: GOM |
| Baudrimont *et al*. (1993) | France | 1 | 59 | F | N/A^b^ | N/A | N/A | Post-mortem: GOM |
| MacLean *et al*. (2005) | USA | 1 | 56 | M | 4 | p.Arg133Cys | gain | Brain biopsy: GOM |
| Ragoschke-Schumm *et al*. (2005) | Germany | 1 | 47 | F | NEG^c^ | NEG | NEG | Skin: GOM |
| Werbrouck and De Bleecker (2006) | Germany | 1 | 45 | M | 4 | p.Arg182Cys | gain | N/A |
| Kotorii *et al*. (2006) | Japan | 1 | 72 | F | 18 | p.Gly975Cys | gain | Skin: NEG |
| Choi *et al*. (2006)^d^ | Korea | 5 | 64.4 (15.4) | M (3)  F (2) | 11  11 | p.Arg544Cys (3)  p.Arg578Cys (1) | gain  gain | N/A  N/A |
| Oh *et al*. (2008) | Korea | 1 | 39 | M | 11 | p.Arg544Cys | gain | N/A |
| Mizuno *et al*. (2008) | Japan | 1 | 64 | F | 3 | p.Arg75Pro | no | Skin: GOM |
| Lee *et al*. (2009) | Taiwan | 4 | 53.5 (5.7) | M (3)  F (1) | 4  11 | p.Arg133Cys (1)  p.Arg544Cys (3) | gain  gain | N/A  N/A |
| Delgado *et al*. (2011) | Spain | 1 | 55 | M | 11 | p.Arg607Cys | gain | N/A |
| Sano *et al*. (2011) | Japan | 1 | 46 | M | 6 | p.Arg332Cys | gain | Skin: GOM |
| Pradotto *et al*. (2012) | Italy | 1 | 65 | M | 13 | p.Arg680Cys | gain | Skin: GOM |
| Lian *et al*. (2013) | China | 1 | 46 | M | 3 | p.Arg90Cys | gain | Skin: GOM |
| Mehta *et al*. (2013) | USA | 1 | 55 | F | 8 | p.Gly420Cys | gain | Skin: inconclusive |
| Rinnoci *et al*. (2013) | Italy | 3 | 67.3 (9.5) | M (2)  F (1) | 14  22 | p.Arg728Cys (1)  p.Arg1231Cys (2) | gain  gain | N/A  N/A |
| Choi *et al*. (2013) | Korea | 4 | 51.8  (14.6) | M (2)  F (2) | 11 | p.Arg544Cys (4) | gain | N/A |
| Marlen *et al*. (2016) | Argentina | 1 | 47 | M | N/A | N/A | N/A | Skin: GOM |
| Koutroulou *et al*. (2016) | Greece | 1 | 30 | M | 4 | p.Arg169Cys | gain | N/A |
| Zhang *et al*. (2017) | China | 2 | 60.5 (6.4) | M (1)  F (1) | 11  13 | p.Arg544Cys (1)  p.Cys681X (1)^e^ | gain  loss | N/A  Skin: GOM |
| Bersano *et al*. (2018) | Italy | 1 | 61 | M | 7 | p.Ser396Cys | gain | N/A |
| Chiang *et al*. (2019) | USA | 1 | 57 | M | N/A^f^ | N/A | N/A | N/A |
| Kim *et al*. (2019) | Korea | 3 | 54 (9.2) | M (3) | 9  11  11 | p.Tyr465Cys (1)  p.Arg54Cys (1)  p.Arg587Cys (1) | gain  gain  gain | N/A  N/A  N/A |
| Wang *et al*. (2020) | China | 1 | 38 | M | 29 | p.Arg1761His | no | Skin: GOM |
| Palazzo *et al*. (2021) | France | 5 | 45.6 (6.2) | M (4)  F (1) | 4  4  12  20 | p.Arg133Cys (2)^g^  p.Arg169Cys  p.Cys617Arg (1)  p.Cys1099Tyr (1) | gain  gain  loss  loss | N/A  N/A  N/A  N/A |
| Liao *et al*. (2021) | Taiwan | 27 | 58.9 (8.2) | M (12)  F (15) | 4  4  5  8  11  11  18 | p.Arg153Cys (1)  p.Cys222Ser (1)  p.Tyr258Cys (1)  p.Arg427Cys (1)  p.Arg544Cys (33)  p.Arg587Cys (1)  p.Cys977Ser (1) | gain  loss  gain  gain  gain  gain  loss | N/A  N/A  N/A  N/A  N/A  N/A  N/A |
| Hu *et al*. (2022) | China | 1 | 60 | M | 11 | p.Arg558Cys | gain | Skin: GOM |
| Chen *et al*. (2022) | Taiwan | 45 | 59.1  (11.6) | M (23)  F (22) | 3  5  7  11  11 | p.Arg75Gln (1)  p.Cys251Trp (1)  p.Arg332Cys (1)  p.Arg544Cys (41)  p.Arg587Cys (1) | no  loss  gain  gain  gain | N/A  N/A  N/A  N/A  N/A |
| Chu *et al*. (2023) | China | 1 | 59 | M | 11 | p.Arg587Cys | gain | N/A |
| Nogueira *et al*. (2023) | Brazil | 1 | 40 | M | 4 | p.Ser145Cys | gain | N/A |

Abbreviation: Arg, arginine; Cys, cysteine; F, female; Gly, glycine; GOM, granular osmiophilic material; His, histidine; M, male; N/A, not applicable; NEG, negative; No., number; Pro, proline; Ser, serine; Tyr, tyrosine

^a^ Age when the patient had ICH was used. Studies with more than one value were shown in mean (SD).

^b^ The first two cases were reported before the NOTCH3 mutation was discovered.

^c^ Genetic testing was done using direct bidirectional sequencing of exons 2-24, which encoded all 34 extracellular EGFr domains.

^d^ Mutation detail was not available in one patient who had CADASIL clinically diagnosed by symptoms, imaging, and being a biological mother of the homozygous patient.

^e^ This patient had a CGCT insertion between nucleotides 2041 and 2042 of exon 13, resulting in cysteine to frameshift amino acid changes at codon 681. A skin biopsy confirmed the diagnosis of CADASIL.

^f^ The patient was reported a genetically confirmed CADASIL, but mutation details were not mentioned in the original paper.

^g^ One of the patients died shortly after the ICH without having genetic testing, and the mutation was revealed 10 years later in his twin daughters.

**Table S7** Summary of characteristics of Asian and non-Asian CADASIL patients with ICH, including cases from our cohort (129 patients)

| **Characteristics**^a^ | Asian (n = 98) | Non-Asian (n = 31) |
| --- | --- | --- |
| Age at ICH; y, mean ± SD | 58.1 ± 15.4 | 51.9 ± 10.6 |
| Male; n (%) | 45 (57.1) | 21 (67.7) |
| Diagnosis of CADASIL; n(%)^b^ |  |  |
| Cysteine-changing *NOTCH3* mutation | 94 (95.9) | 25 (80.6) |
| Cysteine-sparing *NOTCH3* mutation | 3 (3.1) | 0 (0.0%) |
| GOM | 0 (0.0) | 4 (12.9) |
| Known family mutation with clinical features | 1 (1.0) | 1 (3.2) |
| CADASIL feature; n (%) |  |  |
| ICH as the first manifestation | 24/53 (45.3) | 8/31 (25.8) |
| Previous ischaemic stroke | 34/98 (34.7) | 8/31 (25.8) |
| Previous migraine | 0/41 (0.0) | 10/30 (33.3) |
| Previous cognitive impairment | 6/41 (14.3) | 9/30 (30.0) |
| Vascular risk factors; n (%) |  |  |
| Hypertension | 77/98 (78.6) | 14/30 (46.7) |
| Diabetes mellitus | 15/71 (21.1) | 1/30 (3.3) |
| Hyperlipidaemia | 19/71 (19.4) | 7/30 (23.3) |
| History of smoking | 16/71 (22.5) | 8/29 (27.6) |
| Antithrombotic medication; n (%) |  |  |
| Antiplatelet^c^ | 25/48 (52.1) | 12/29 (41.4) |
| Aspirin | 13/20 (65.0) | 9/12 (75.0) |
| Clopidogrel | 4/20 (20.0) | 3/12 (25.0) |
| Anticoagulant | 1/48 (2.3) | 5/29 (17.2) |
| Warfarin | 1/1 (100.0) | 2/3 (66.7) |
| LMWH | 0/1 (0.0) | 1/3 (33.3) |
| Symptom; n (%) |  |  |
| Headache | 1/8 (12.5) | 11/29 (37.9) |
| Altered mental status | 2/8 (25.0) | 11/29 (37.9) |
| Weakness | 4/8 (50.0) | 18/29 (62.1) |
| Dysphasia | 2/8 (25.0) | 8/29 (27.6) |
| Seizure | 2/8 (25.0) | 2/29 (34.5) |
| Recurrent ICH | 14/91 (15.4) | 2/31 (6.5) |
| Intraventricular extension | 15/91 (16.5) | 10/25 (40.0) |
| Died from ICH | 4/98 (4.1) | 7/31 (22.6) |
| ICH location (142 lesions); n (%)^d^ |  |  |
| Thalamus | 48/109 (44.4) | 10/33 (30.3) |
| Basal ganglia | 27/109 (24.8) | 7/33 (21.2) |
| Subcortical WM | 13/109 (11.9) | 5/33 (15.2) |
| Cortical | 5/109 (4.6) | 5/33 (15.2) |
| Cerebellum | 8/109 (7.3) | 4/33 (12.1) |
| Brainstem | 6/109 (5.5) | 2/33 (6.1) |
| Imaging characteristics; n (%) |  |  |
| Have CMB | 79/80 (98.8) | 20/25 (80.0) |
| CMBs ≥ 10 | 58/64 (90.6) | 8/20 (40.0) |
| Have Lacune | 64/68 (94.1) | 22/25 (88.0) |

Abbreviation: CADASIL, cerebral autosomal dominant arteriopathy with subcortical infarcts and leukoencephalopathy; CMB, cerebral microbleed; ICH, intracerebral haemorrhage

^a^Due to a variety of reported individual-level patient data in different studies, the denominators differed in each characteristic.

^b^One patient had cysteine-sparing mutation without other details. Another patient was reported genetically confirmed CADASIL, but the mutation was not mentioned.

^c^Other antiplatelets included cilostazol in 1 patient (3.1%), aspirin and dipyridamole in 1 patient (3.1%), and ticlopidine in 1 patient (3.1%).

^d^The exact location was not known in 2 cases. One case reported a parietal lobe ICH, but the exact region wasn’t mentioned. Another case reported multiple ICH lesions.
